# Supplementary figures and images for: Inflammatory state of lymphatic vessels and miRNA profiles associated with relapse in ovarian cancer patients
Source: PLoS One. 2020 Jul 27;15(7):e0230092. doi: 10.1371/journal.pone.0230092 (PMC7384632; doi:10.1371/journal.pone.0230092)

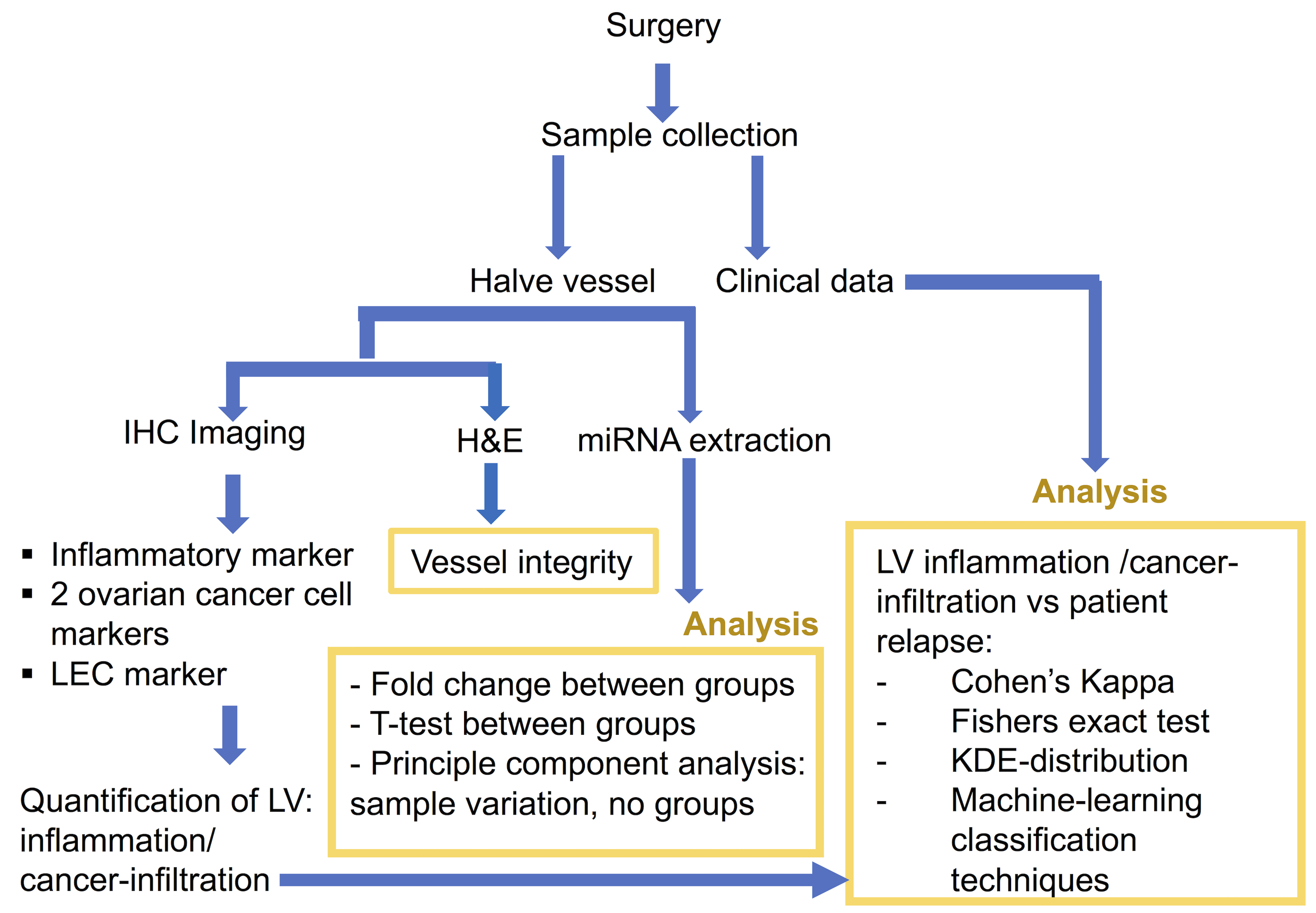

Supplement: S1 Fig — Each vessel dissected from one sample is divided into 2 and processed for IHC and miRNA expression analysis. (JPEG) [file pone.0230092.s002.jpeg]

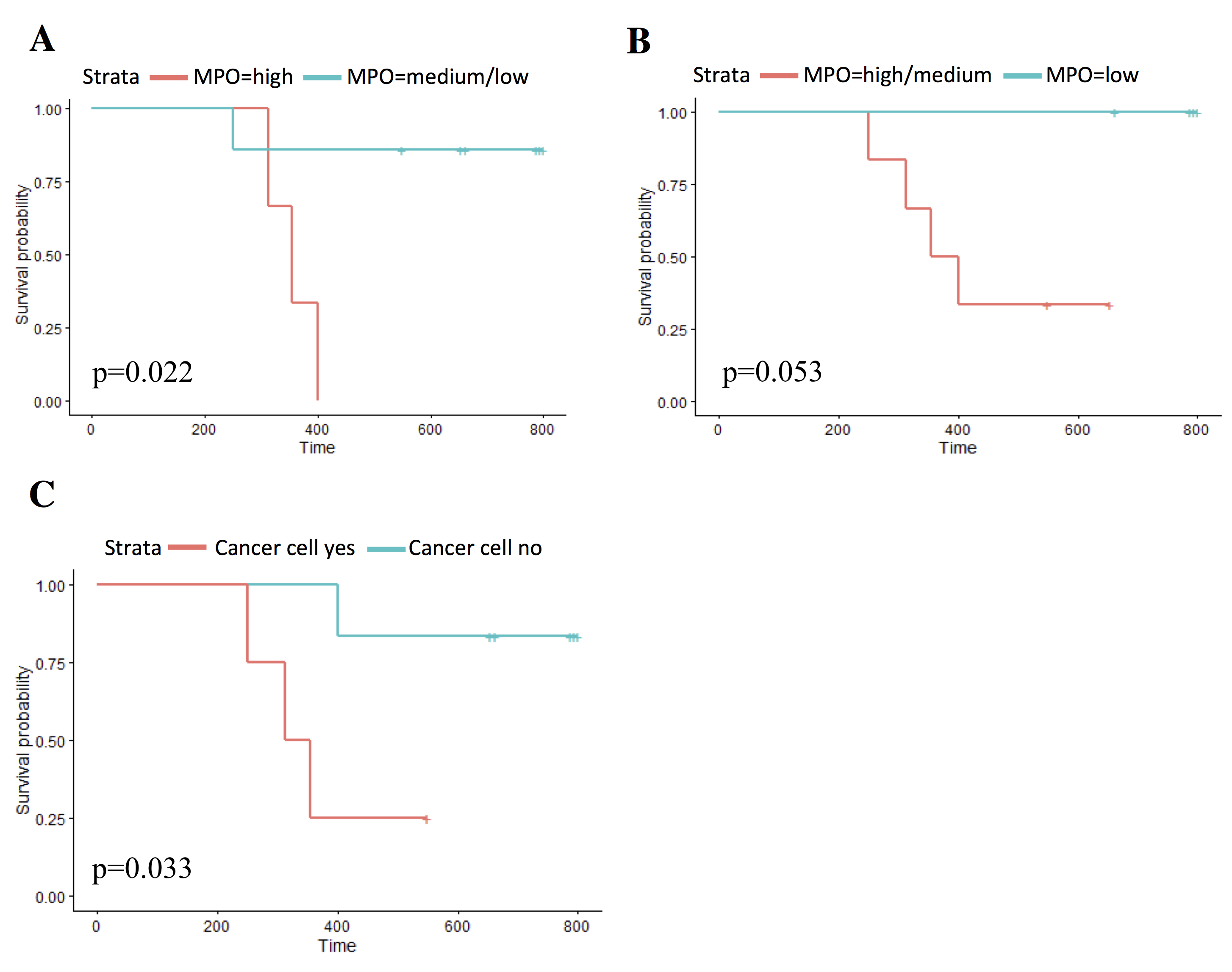

Supplement: S2 Fig — A. According to inflammation (MPO) (high vs. medium/low) B. According to inflammation (MPO) (high/medium vs. low). C. According to cancer cell infiltration (WT1 or Pax.8). (TIF) [file pone.0230092.s003.tif]

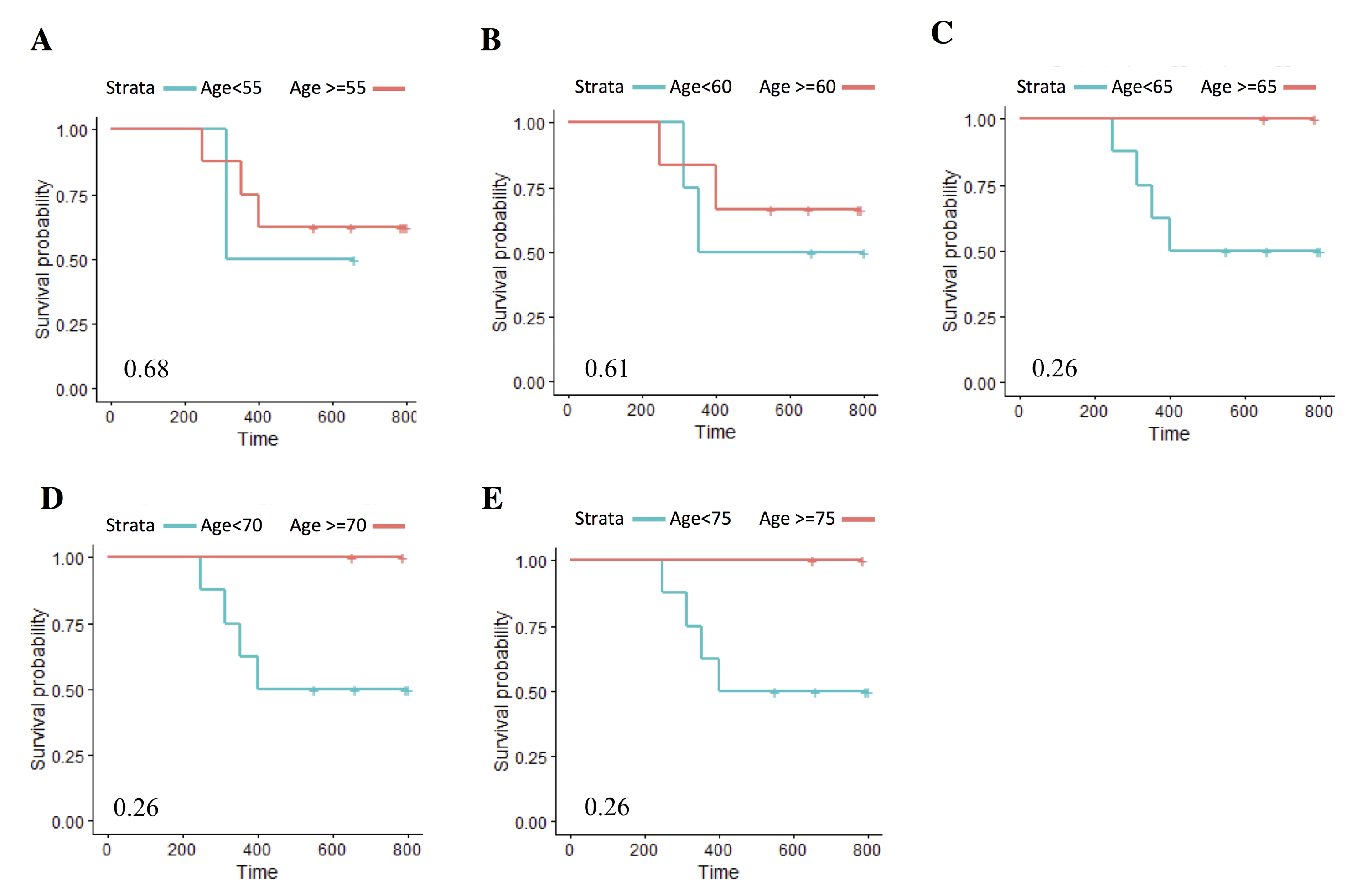

Supplement: S3 Fig — A. Under 55 years B. Under 60 years C. Under 65 years. D Under 70years. E Under 75years. (TIF) [file pone.0230092.s004.tif]

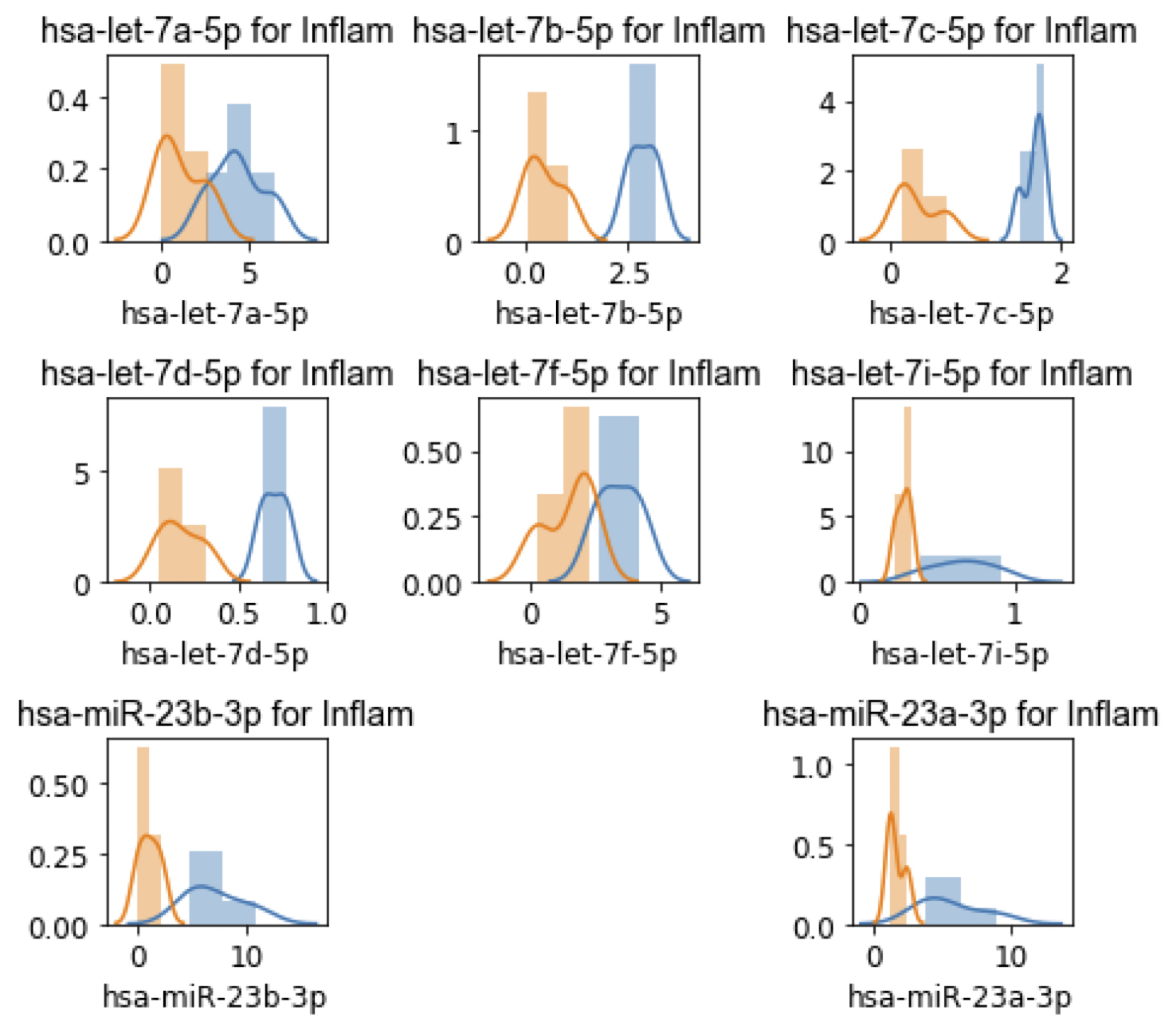

Supplement: S4 Fig — (JPEG) [file pone.0230092.s005.jpeg]
