# Supplementary material for: Inflammatory state of lymphatic vessels and miRNA profiles associated with relapse in ovarian cancer patients
Source: PLoS One. 2020 Jul 27;15(7):e0230092. doi: 10.1371/journal.pone.0230092 (PMC7384632; doi:10.1371/journal.pone.0230092)

A

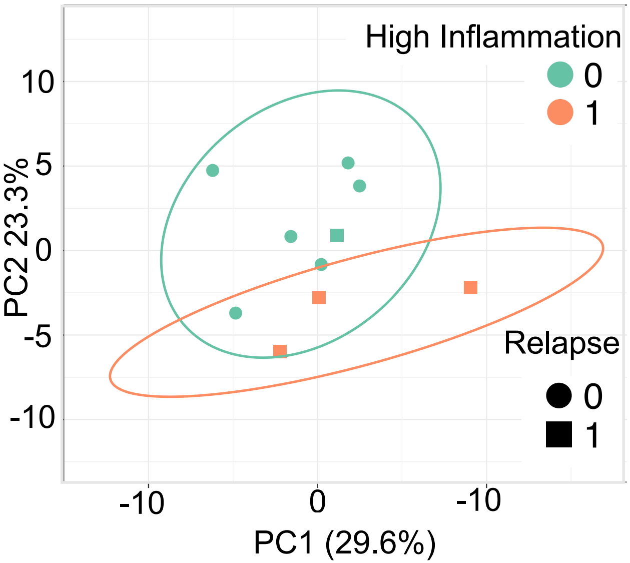

B

PC1		PC2	
**miR-29b-3p**	-0.222	miR-125b-5p	0.243
miR-186-5p	-0.211	miR-98-5p	0.243
**miR-301a-3p**	-0.210	miR-125a-5p	0.221
**miR-101-3p**	-0.205	miR-17-5p	0.216
**miR-19b-3p**	-0.195	miR-30a-5p	0.202
**miR-181c-5p***	-0.194	**miR-23a-3p***	0.200
**miR-19a-3p**	-0.192	miR-15b-5p	0.198
miR-30b-5p	-0.192	**let-7b-5p***	0.196
**miR-497-5p***	-0.191	**let-7i-5p***	0.194
miR-145-5p	-0.189	miR-20b-5p	0.188
**miR-16-5p***	-0.186	**let-7d-5p***	0.187
**miR-15a-5p**	-0.184	miR-340-5p	0.183
miR-34a-5p	-0.171	**let-7c-5p***	0.178
miR-424-5p	-0.171	**let-7a-5p***	0.171
miR-20a-5p	-0.170	miR-20a-5p	0.168
miR-128-3p	-0.165	miR-545-3p	0.165
miR-30d-5p	-0.155	**let-7f-5p***	0.151
miR-29c-3p	-0.150	miR-30e-5p	0.149
**let-7d-5p***	0.146	miR-181b-5p	0.147
**let-7c-5p***	0.143	**let-7e-5p***	0.145

C

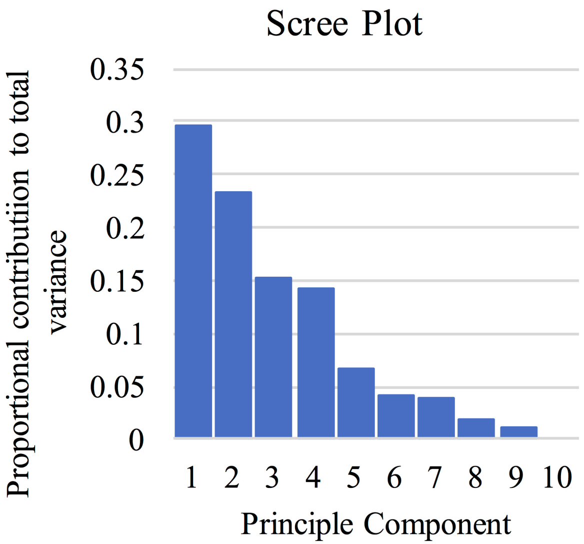

Supplement: S5 Fig — A. Principle component analysis showed that inflamed and non-inflamed samples show variance across Principle Component (PC) 2. Samples with most similar miRNA expression profile cluster together. Unit variance scaling was applied to rows; SVD with imputation was used to calculate principal components. Prediction ellipses are such that with probability 0.95, a new observation from the same group will fall inside the ellipse. N = 10 data points. Figures produced with ClustVis [26] B. The miRNA expression driving PC1 & 2 contributed to the greatest variation between sample groups with several miRNA identified as differentially regulated when comparing inflamed or cancer-infiltrated LVs (bold = >±1.8, * = p<0.05). C. A Scree plot showing the amount of variation described by each PC confirmed that the two primary PCs accounted for much of the variation between all sample. (PDF) [file pone.0230092.s006.pdf]
