# Supplementary material for: Inflammatory state of lymphatic vessels and miRNA profiles associated with relapse in ovarian cancer patients
Source: PLoS One. 2020 Jul 27;15(7):e0230092. doi: 10.1371/journal.pone.0230092 (PMC7384632; doi:10.1371/journal.pone.0230092)
Supplement: S1 Table — (PDF) [file pone.0230092.s007.pdf]

Sample	Age	Surgery	Residual Disease	LN sampled	LN histology
1	54	Primary	nil	Right pelvic	negative
2	57	Primary	nil	No	n/a
3	62	Interval	nil	No	n/a
4	64	Interval	nil	No	n/a
5	57	Primary	nil	No	n/a
6	61	Interval	nil	No	n/a
7	81	primary	nil	Para-aortical	positive
8	41	Interval	nil	No	n/a
9	75	Primary	nil	No	n/a
10	64	primary	nil	Left pelvic &para-aortic	both negative
