# Supplementary material for: Inflammatory state of lymphatic vessels and miRNA profiles associated with relapse in ovarian cancer patients
Source: PLoS One. 2020 Jul 27;15(7):e0230092. doi: 10.1371/journal.pone.0230092 (PMC7384632; doi:10.1371/journal.pone.0230092)
Supplement: S2 Table — We compared expression in LVs with high versus low inflammation (n = 7) Listed are miRNA that showed a fold-regulation change ±1.8 with those showing a significant difference between groups highlighted (t-test p>0.05). * = miRNA that remained below a Bonferroni correction of p<0.00208. (PDF) [file pone.0230092.s008.pdf]

LVs with high versus low inflammation

miRNA	Fold Regulation	p value
miR-301a-3p	5.766	0.4688
miR-144-3p	4.173	0.1242
miR-186-5p	4.138	0.1288
miR-19a-3p	3.953	0.3073
miR-19b-3p	3.456	0.2669
miR-101-3p	3.075	0.2711
miR-15a-5p	3.011	0.2785
miR-410-3p	2.401	0.8776
miR-497-5p	2.319	0.2108
miR-548e-3p	-2.064	0.4127
miR-125a-5p	-2.200	0.1220
**let-7i-5p**	**-2.233**	**0.0328**
let-7e-5p	-2.666	0.0802
let-7g-5p	-2.986	0.0969
**let-7f-5p**	**-3.198**	**0.0378**
**miR-23a-3p**	**-3.443**	**0.0401**
miR-545-3p	-3.473	0.2340
miR-98-5p	-4.635	0.0823
**let-7d-5p ***	**-5.057**	**0.0009**
miR-15b-5p	-5.238	0.0696
miR-181d-5p	-5.972	0.1116
miR-130a-3p	-6.144	0.7502
**let-7c-5p ***	**-6.425**	**0.0003**
miR-607	-7.332	0.1901
miR-202-3p	-7.821	0.1220
miR-656-3p	-8.224	0.2351
**let-7b-5p ***	**-11.423**	**0.0006**
**miR-23b-3p**	**-15.166**	**0.0152**
**hsa-let-7a-5p**	**-188.204**	**0.0389**

up-regulated	down-regulated	**Bold= p < 0.05**
