# Supplementary material for: Inflammatory state of lymphatic vessels and miRNA profiles associated with relapse in ovarian cancer patients
Source: PLoS One. 2020 Jul 27;15(7):e0230092. doi: 10.1371/journal.pone.0230092 (PMC7384632; doi:10.1371/journal.pone.0230092)
Supplement: S3 Table — We compared expression in LVs with high or medium inflammation versus low inflammation (n = 10) Listed are miRNA that showed a fold-regulation change ±1.8 with those showing a significant difference between groups highlighted (t-test p>0.05). * = miRNA that remained below a Bonferroni correction of p<0.00161. (PDF) [file pone.0230092.s009.pdf]

LVs with low versus [medium or high] inflammation		
miRNA	Fold regulation	p-value
miR-410-3p	10.146	0.442
miR-144-3p	4.022	0.104
miR-301a-3p	3.501	0.725
miR-101-3p	2.700	0.368
miR-381-3p	2.408	0.170
miR-19a-3p	2.306	0.472
miR-497-5p	2.168	0.168
miR-19b-3p	2.028	0.422
miR-181c-5p	2.019	0.186
miR-186-5p	2.000	0.408
miR-15a-5p	1.888	0.452
miR-340-5p	-1.819	0.217
miR-98-5p	-1.843	0.427
miR-454-3p	-1.909	0.339
miR-181d-5p	-1.938	0.987
miR-130a-3p	-1.951	0.589
**let-7i-5p**	**-2.021**	**0.013**
miR-374a-5p	-2.109	0.228
miR-15b-5p	-2.154	0.268
let-7d-5p	-2.291	0.566
**let-7g-5p**	**-2.390**	**0.031**
miR-202-3p	-2.497	0.682
miR-607	-2.578	0.523
miR-656-3p	-2.741	0.600
miR-545-3p	-2.773	0.074
let-7c-5p	-2.982	0.347
miR-23b-3p	-3.654	0.406
let-7b-5p	-4.443	0.336
let-7a-5p	-14.817	0.793

up-regulated	down-regulated	**Bold= $p < 0.05$**
