# Supplementary material for: Inflammatory state of lymphatic vessels and miRNA profiles associated with relapse in ovarian cancer patients
Source: PLoS One. 2020 Jul 27;15(7):e0230092. doi: 10.1371/journal.pone.0230092 (PMC7384632; doi:10.1371/journal.pone.0230092)
Supplement: S4 Table — Listed are miRNA that showed a fold-regulation change ±1.8 with those showing a significant difference between groups highlighted (t-test p>0.05). * = miRNA that remained below a Bonferroni correction of p<0.002. (PDF) [file pone.0230092.s010.pdf]

Cancer-infiltrated vs non-cancer-infiltrated LVs

miRNA	Fold Regulation	p value
**miR-144-3p***	**14.020**	**0.0010**
**miR-181c-5p**	**10.629**	**0.0170**
miR-301a-3p	7.300	0.3090
miR-19a-3p	6.896	0.1970
miR-19b-3p	6.055	0.1470
miR-101-3p	5.083	0.0740
**miR-381-3p**	**4.070**	**0.0140**
miR-181a-5p	3.875	0.2740
miR-130a-3p	3.853	0.1150
miR-186-5p	3.214	0.1310
let-7a-5p	2.920	0.2870
**miR-497-5p**	**2.902**	**0.0260**
miR-590-5p	2.780	0.5210
miR-15a-5p	2.767	0.1820
**miR-93-5p***	**2.252**	**0.0010**
miR-29a-3p	2.166	0.5250
miR-29b-3p	2.098	0.3200
**miR-16-5p**	**2.077**	**0.0310**
**let-7i-5p**	**-2.080**	**0.0210**
let-7b-5p	-2.274	0.0700
let-7e-5p	-2.505	0.4320
let-7c-5p	-2.556	0.0780
let-7d-5p	-2.672	0.1220
let-7f-5p	-3.239	0.2700
miR-202-3p	-4.800	0.2990

up-regulated	down-regulated	**Bold= p < 0.05**
