# Supplementary material for: Inflammatory state of lymphatic vessels and miRNA profiles associated with relapse in ovarian cancer patients
Source: PLoS One. 2020 Jul 27;15(7):e0230092. doi: 10.1371/journal.pone.0230092 (PMC7384632; doi:10.1371/journal.pone.0230092)
Supplement: S5 Table — Listed are miRNA that showed a fold-regulation change ±1.8 with those showing a significant difference between groups highlighted (t-test p>0.05). Bonferroni correction of p<0.002. (PDF) [file pone.0230092.s011.pdf]

LVs from relapse versus non-relapse patients

miRNA	Fold regulation	p-value
miR-144-3p	5.069	0.0906
**miR-186-5p**	**4.930**	**0.0294**
miR-301a-3p	3.750	0.4580
miR-19b-3p	3.267	0.2037
miR-19a-3p	3.183	0.2633
miR-15a-5p	2.975	0.1666
miR-29b-3p	2.609	0.1861
miR-29c-3p	2.475	0.4066
miR-497-5p	2.295	0.0672
miR-101-3p	2.117	0.2561
miR-34a-5p	2.089	0.0828
miR-548d-3p	2.009	0.6136
miR-30e-5p	-1.827	0.3177
miR-548e-3p	-1.917	0.2868
let-7g-5p	-1.938	0.1780
let-7f-5p	-1.954	0.4330
miR-23a-3p	-2.769	0.1140
miR-449a	-3.197	0.3953
miR-15b-5p	-3.302	0.1104
miR-98-5p	-3.430	0.0673
let-7d-5p	-3.681	0.0645
miR-181d-5p	-4.029	0.1767
miR-130a-3p	-4.306	0.5101
**let-7c-5p**	**-4.335**	**0.0385**
miR-301b-3p	-4.776	0.1515
**let-7b-5p**	**-6.940**	**0.0383**
**miR-23b-3p**	**-8.529**	**0.0278**
miR-656-3p	-16.377	0.3143
let-7a-5p	-42.416	0.2375

up-regulated | down-regulated | **Bold= p < 0.05**
