# Supplementary material for: Inflammatory state of lymphatic vessels and miRNA profiles associated with relapse in ovarian cancer patients
Source: PLoS One. 2020 Jul 27;15(7):e0230092. doi: 10.1371/journal.pone.0230092 (PMC7384632; doi:10.1371/journal.pone.0230092)
Supplement: S6 Table — Depicted is the accuracy of the algorithm to predict LV inflammation or patient relapse (percentage of correct predictions), the per-group accuracy (No Inflam., Yes Inflam. / Stage < IV, Stage > = IV), Cohen’s Kappa and thus the agreement between predicted and actual states, and McNemar’s test significance of equality of predicted probability (inner accuracy) between groups for each outcome. (PDF) [file pone.0230092.s012.pdf]

Down-regulated miRNA			
KEGG pathway	p-value	Genes	miRNA
Tarbase			
Proteoglycans in cancer	1.86E-11	88	3
Viral carcinogenesis	4.65E-08	77	3
Fatty acid biosynthesis	9.08E-08	4	3
Cell cycle	9.08E-08	58	3
Lysine degradation	1.42E-07	22	3
Protein processing in endoplasmic reticulum	1.56E-06	72	3
Adherens junction	1.64E-05	37	3
p53 signaling pathway	2.94E-05	36	3
Epstein-Barr virus infection	8.24E-05	84	3
TGF-beta signaling pathway	1.47E-04	33	3
Hippo signaling pathway	1.47E-04	55	3
FoxO signaling pathway	1.49E-04	58	3
Chronic myeloid leukemia	3.27E-04	33	3
Focal adhesion	1.12E-03	80	3
TargetScan			
Mucin type O-Glycan biosynthesis	1.78E-04	1	2
Valine, leucine and isoleucine biosynthesis	5.71E-04	1	2
Signaling pathways regulating pluripotency of stem cells	1.67E-02	6	2
2-Oxocarboxylic acid metabolism	2.07E-02	1	2
Valine, leucine and isoleucine degradation	2.58E-02	2	3
Biosynthesis of amino acids	3.21E-02	2	2
Micro-CT-DS			
ECM-receptor interaction	3.00E-06	19	4
Signaling pathways regulating pluripotency of stem cells	3.00E-06	47	4
TGF-beta signaling pathway	3.20E-06	28	4
ErbB signaling pathway	1.32E-05	35	4
Long-term potentiation	1.85E-04	27	4
Proteoglycans in cancer	1.85E-04	58	4
Mucin type O-Glycan biosynthesis	3.89E-04	9	4
Axon guidance	4.95E-04	37	4
mTOR signaling pathway	9.62E-04	24	4
Adrenergic signaling in cardiomyocytes	1.03E-03	41	4
FoxO signaling pathway	1.05E-03	41	4
Glutamatergic synapse	1.31E-03	32	4
Focal adhesion	1.88E-03	57	4
