# Supplementary material for: Inflammatory state of lymphatic vessels and miRNA profiles associated with relapse in ovarian cancer patients
Source: PLoS One. 2020 Jul 27;15(7):e0230092. doi: 10.1371/journal.pone.0230092 (PMC7384632; doi:10.1371/journal.pone.0230092)
Supplement: S7 Table — Accuracy, Cohen’s Kappa, Mc Nemar p-value of equality for inner grouped probabilities and classification are reported for the predicted classes. The expression of the most significantly differentially expressed miRNA were added one by one, or in pairs if significance was equal, into the parameter set used to build the classifiers. When only significantly differentially expressed miRNA identified in inflamed LVs (high and medium inflammation versus low) were used to build the classifier, the accuracy of subsequent predictions increased by 20–40% compared to classifiers based on all available miRNA expression (S6 Table). Similar improvements were found in the accuracy of classifiers predicting relapse and LV cancer-infiltration (Tables 7 and 8). (PDF) [file pone.0230092.s013.pdf]

Method	Accuracy	Inflammation<br>McNemar's Test-pval	Cohen's Kappa	No Inflam	Yes Inflam
**Logistic Regression**	40%	0.61	-0.25	25.0%	50.0%
**K-Nearest Neighbours**	50%	1	-0.04	75.0%	33.3%
**Support Vector Machine**	60%	0.24	0.24	0.0%	50.0%
**Random Forests Classifier**	30%	0.68	0.16	50.0%	67.0%
**Gaussian Naive Bayes**	50%	0.07	-0.04	0.0%	83.3%

Method	Accuracy	Stage>=IV<br>McNemar's Test-pval	Cohen's Kappa	Stage <IV	Stage >=IV
**Logistic Regression**	40%	0.61	-0.25	25.0%	50.0%
**K-Nearest Neighbours**	60%	0.22	0.16	83.3%	25.0%
**Support Vector Machine**	60%	0.04	0.16	100.0%	0.0%
**Random Forests Classifier**	30%	1	-0.45	33.3%	25.0%
**Gaussian Naive Bayes**	50%	0.007	-0.04	83.3%	0.0%
