# Supplementary material for: Inflammatory state of lymphatic vessels and miRNA profiles associated with relapse in ovarian cancer patients
Source: PLoS One. 2020 Jul 27;15(7):e0230092. doi: 10.1371/journal.pone.0230092 (PMC7384632; doi:10.1371/journal.pone.0230092)
Supplement: S8 Table — (PDF) [file pone.0230092.s014.pdf]

Inflam	Method	Accuracy	Cohen's Kappa	No Inflam	Inflam	Mc Nemar p-value
**1-St.Sig miRNAs**	Logistic Regression	60	0.17	0	100	0.04
	K-Nearest Neighbours	80	0.58	75	83.3	0.72
	Support Vector Machines	60	0.17	0	100	0.04
	Random Forests	70	0.37	50	83.3	0.45
	Gaussian Naive Bayes	80	0.58	75	83.3	0.72
**2- St.Sig miRNAs**	Logistic Regression	70	0.37	50	83.3	0.45
	K-Nearest Neighbours	80	0.58	50	100	0.29
	SVM	80	0.58	50	100	0.29
	Random Forests	70	0.37	50	83.3	0.45
	Gaussian Naive Bayes	80	0.58	75	83.3	0.72

Relapse	Method	Accuracy	Cohen's Kappa	No Relapse	Relapse	Mc Nemar p-value
**2-St.Sig miRNAs**	Logistic Regression	80	0.58	100	50	0.29
	K-Nearest Neighbours	90	0.79	100	75	0.5
	Support Vector Machines	80	0.58	83.3	75	0.72
	Random Forests	60	0.16	67	50	0.68
	Gaussian Naive Bayes	80	0.28	100	50	0.58
**4-St.Sig miRNAs**	Logistic Regression	70	0.37	83.3	50	0.45
	K-Nearest Neighbours	90	0.79	100	75	0.5
	Support Vector Machines	90	0.79	100	75	0.5
	Random Forests	60	0.16	67	50	0.68
	Gaussian Naive Bayes	80	0.28	100	50	0.58

Cancer	Method	Accuracy	Cohen's Kappa	No Cancer	Cancer	Mc Nemar p-value
**2-St.Sig, miRNAs**	Logistic Regression	60	0.17	100	0	0.04
	K-Nearest Neighbours	100	1	100	100	1
	Support Vector Machine	60	0.17	100	0	0.04
	Random Forests	100	1	100	100	1
	Gaussian Naive Bayes	100	1	100	100	1
**3-St.Sig, miRNAs**	Logistic Regression	60	0.17	100	0	0.04
	K-Nearest Neighbours	100	1	100	100	1
	Support Vector Machines	60	0.17	100	0	0.04
	Random Forests	100	1	100	100	1
	Gaussian Naive Bayes	100	1	100	100	1
**5-St.Sig, miRNAs**	Logistic Regression	60	0.17	100	0	0.04
	K-Nearest Neighbours	100	1	100	100	1
	Support Vector Machines	60	0.17	100	0	0.04
	Random Forests	100	1	100	100	1
	Gaussian Naive Bayes	100	1	100	100	1
**6-St.Sig, miRNAs**	Logistic Regression	70	0.37	83.3	50	0.45
	K-Nearest Neighbours	90	0.79	83	100	1
	Support Vector Machines	100	1	100	100	1
	Random Forests	100	1	100	100	1
	Gaussian Naive Bayes	100	1	100	100	1
**7-St.Sig, miRNAs**	Logistic Regression	80	0.58	100	50	0.45
	K-Nearest Neighbours	100	1	100	100	1
	SVM	100	1	100	100	1
	Random Forests	100	1	100	100	1
	Gaussian Naive Bayes	100	1	100	100	1
